# High-Density Lipoproteins and Cardiovascular Disease: The Good, the Bad and the Future

**DOI:** 10.3390/biomedicines9080857

**Published:** 2021-07-22

**Authors:** Josep Julve, Joan Carles Escolà-Gil

**Affiliations:** 1 Institut d’Investigacions Biomèdiques IIB Sant Pau, 08041 Barcelona, Spain; 2 CIBER de Diabetes y Enfermedades Metabólicas Asociadas (CIBERDEM), 28029 Madrid, Spain; 3 Departament de Bioquímica i Biologia Molecular, Universitat Autònoma de Barcelona, 08041 Barcelona, Spain

Epidemiological studies have shown that low levels of plasma high-density lipoprotein cholesterol (HDL-C) are associated with increased atherosclerotic cardiovascular disease (CVD). However, accumulating experimental evidence has challenged this epidemiologic notion in the last decade, since HDL-C-raising strategies failed to confer increased cardioprotection in subjects at high risk for cardiovascular disease [1,2]. Thus, it became evident that HDL composition and function, rather than simply the plasma concentrations HDL-C levels, should be targeted to prevent future cardiovascular events. Circulating HDL are currently considered as complex macromolecules with several bioactive properties and a favorable influence on several biological processes, some of them with a marked anti-atherogenic footprint. The function of this class of lipoproteins is strongly determined by a plethora of proteins and lipids. Because an impaired HDL remodeling and metabolism may influence their composition, those components could be used as potential therapeutic targets to improve HDL functionality [3]. In this context, the clinical significance of HDL in different physio-pathological scenarios were debated in the Special Issue “High-Density Lipoproteins and Cardiovascular Disease: The Good, the Bad, and the Future” of the *Biomedicines* journal. The mechanisms of HDL modifications and their functional implications was exhaustively reviewed by Márquez et al. [3]. Other studies published in this Special Issue also demonstrated the predictive value by HDL-mediated cholesterol efflux in subjects at high risk for cardiovascular mortality [4] and subjects with metabolically-driven non-alcoholic fatty liver disease (NAFLD) [5], which is considered the hepatic component of metabolic syndrome, and thus associated with an increased risk of atherosclerotic cardiovascular disease [6].

Beyond the predictive value of compositional and functional properties of HDL, a retrospective study also revealed the role of low HDL-C in predicting the risk of developing coronary artery ectasia in healthy subjects [7]. In line with this study, plasma concentrations of HDL-C were significantly decreased in subjects with familial combined hyperlipidemia (FCH), one of the most prevalent proatherogenic dyslipemias in humans, as compared with healthy non-FCH subjects, regardless of triglyceridemia [8]. In this study, the HDL-C levels remained lower than those in healthy controls, even after statin treatment. Consistently, the HDL-mediated ability to stimulate cholesterol efflux and to protect LDL from oxidation was similar in both normo- and hyper-triglyceridemic FCH subjects, regardless of statin treatment, as compared with healthy non-FCH controls [8]; however, the relative proportion of the lipoprotein(Lp)-associated phospholipase A2 (Lp-PLA2) in HDL reached values of non-FCH subjects in both groups of subjects treated with statins. Although the biological significance of the relative increase in this HDL-associated enzyme was not further explored, it could have a role in the modulation of the inflammatory response and chemotaxis/adhesion of immune cells in target tissues [9]. Potentially, the findings by Puig et al. [8] would potentially uncover a novel HDL-related biomarker for FCH prognosis.

The clinical significance of HDL changes in composition or function do not only relate to atherosclerosis. Dysfunctional HDL has also been associated with other diseases, including cognitive impairment [10,11] and allergy and skin diseases [9].

Recent findings from epidemiological studies suggest an inverse relationship between HDL-cholesterol (HDL-C) and Alzheimer’s disease (AD). In the context of neurological disabilities, accumulating experimental evidence supports an HDL-mediated protection against memory deficits, neuroinflammation, and endothelial dysfunction [12]. However, the potential use of functional HDL characteristics for the diagnosis of AD still remains poorly explored. In this context, two of the reports published in this Special Issue were conducted to directly assess the contribution of quantitative characteristics of circulating HDL in disabling cerebral beta-amyloidosis pathologies, such as AD [10,11]. Interestingly, the HDL isolated from AD subjects were more electronegative than those from non-AD subjects [11], possibly suggesting the presence of lipoproteins with enhanced bioactive properties. Consistently, the HDL isolated from plasma of AD subjects were more proinflammatory and, interestingly, had less ability to stimulate cholesterol efflux. These findings hence showed that the HDL from AD subjects would exert less atheroprotection than those of non-AD. Noteworthy, the most electronegative HDL subfraction from plasma HDL of AD subjects had a relatively higher content of lipids and ApoC-III than that from non-AD [11]. Intriguingly, the relative content of ApoC-III in plasma HDL of AD subjects did not differ from that of non-AD subjects in the study of Bonaterra-Pastra et al. [10], being, though marginally, decreased rather than increased. The rationale for such controversy is unknown, but it could be, at least in part, due to the fact that the different groups of the latter study were gender- and age-matched [10], whereas the study from Chan et al. were not [11]. Additionally, in the study of Bonaterra-Pastra et al. [10] relative apolipoprotein composition of HDL was also analyzed in subjects with diagnosed cerebral amyloid angiopathy (CAA) due to lobar intracerebral hemorraghe and compared with matched healthy controls. Interestingly, data revealed alterations in the HDL composition, as revealed by relative reductions in the ApoC-III of HDL isolated from subjects with CAA. Reduced HDL-ApoC-III was accompanied by an increased content of cholesterol, mainly due to its esterified form, in this lipoprotein class. Overall, data from the latter study strongly suggested a differential remodeling of HDL in subjects with diagnosed CAA compared with AD and could help to a differential diagnosis of both outcomes of amyloid deposition in brain vessels. However, neither the mechanisms underlying the relative elevations of cholesterol in HDL of CCA nor the contribution of CAA HDL on vascular dysfunction were directly analyzed in the latter study. Thus, further research is warranted in this field.

Apart from traditionally assigned cardioprotective favorable functions of HDL (i.e., cholesterol efflux, anti-inflammatory, anti-oxidative, anti-thrombotic and anti-apoptotic actions), accumulating data suggest that HDL is also involved in host defence as part of immune system (reviewed in [9]). In line with this, lower plasma concentrations of HDL-C has been observed in allergic asthma and rhinitis, but also in dermatological manifestations commonly associated with an adverse immune response, such as atopic dermatitis (eczema), psoriasis, urticaria, and angiodema [9]. In this interesting review, Trakaki & Marsche clearly dissect the evidence supporting a favorable action by HDL-associated proteins (i.e., ApoA-I, ApoA-IV, and ApoC-III) and lipids (i.e., lysophosphatidylcholines) in modulating the immune response in these conditions. The authors also describe how both allergies and skin diseases negatively influence HDL composition, metabolism and function, and the potential contribution of distorted HDL on disease progression.

An impaired insulin signaling is frequently associated with NAFLD and increased risk of cardiovascular disease [6]. Plasma HDL are frequently dysfunctional in subjects with type 2 diabetes mellitus (T2D) [13]. Currently, the lack of appropriate animal models mimicking characteristics commonly associated with T2D in humans, i.e., a distorted (atherogenic) lipid profile and NAFLD, limit, at least in part, the analysis of the contribution of dysfunctional HDL in these adverse cardiometabolic contexts. In this Special Issue, Khadke et al. [14] reported the development of an animal (rat) model of insulin resistance/T2D that reproduced the atherogenic lipid profile, i.e., elevated LDL-cholesterol and triglycerides and reduced HDL-C, along with signs of NAFLD. Interestingly, NAFLD in diabetic rats were revealed by histological signs of lipid accumulation in histological preparations of hepatic tissue and an increased gene expression of targets of lipogenesis and inflammation. However, the analysis of HDL composition and function was pending in that report. Similarly to the rat model, the metabolically-driven NAFLD subjects were more insulin resistant than healthy non-NAFLD subjects, but whether the ability of insulin-resistant rat HDL to promote cholesterol efflux from macrophages in vitro parallels that of metabolically-driven NAFLD subjects [5] was not addressed in the study by Khadke et al. [14]. If confirmed, it would help to test the impact of novel therapies to manage adverse consequences on HDL function by insulin resistance/T2D.

Finally, the work reported by Cedó et al. [15] was a clear representative of a translational approach. In this study, the administration of virgin olive oil enriched with phenolic compounds promoted the HDL-mediated macrophage cholesterol efflux in vitro. Cholesterol efflux is considered as an attempt to estimate of HDL function. However, it is just the first step of macrophage-specific reverse cholesterol transport (mRCT) [16]. The favorable influence of phenolic compound supplementation of olive oil raised both plasma HDL-C and HDL-specific cholesterol efflux in mice confirmed data from previous studies [17,18]. Because the analysis of the whole mRCT process is not viable in humans, the effect of phenolic extract used in functional phenol-enriched olive oil preparations was directly studied in mice in the study by Cedó et al. [15]. Importantly, data showed that the administration of phenolic extract stimulated the whole mRCT pathway, thereby providing for the first time, evidence of the crucial role of phenolic compounds in the induction of mRCT in vivo.

In summary, studies published in this Special Issue provided clinical evidence of the usefulness of alterations in HDL composition and function in a wide spectra of diseases and helped define novel targets for the diagnosis/prognosis/therapeutics of cardiovascular/neurological/autoimmune diseases.
